# 1762. A Review of Antibiotic Outcomes Data Utilizing the Multidisciplinary OPTIONS-DC Conference for PWUD

**DOI:** 10.1093/ofid/ofac492.1392

**Published:** 2022-12-15

**Authors:** Alyse H Douglass, Heather Mayer, Kathleen Young, Amber C Streifel, Heather Franklin, Luke Strnad, Jina T Makadia, Monica K Sikka

**Affiliations:** Oregon Health and Science University, Portland, OR; Oregon Health and Science University, Portland, OR; Oregon Health & Science University, Tigard, Oregon; Oregon Health and Science University, Portland, OR; Oregon Health & Sciences University, Portland, Oregon; Oregon Health and Science University, Portland, OR; Oregon Health and Science University, Portland, OR; Oregon Health and Science University, Portland, OR

## Abstract

**Background:**

Persons who use drugs (PWUD) admitted for serious infections face many challenges when long courses of intravenous (IV) antibiotics are recommended. OPTIONS-DC is a structured multidisciplinary care conference used to navigate some of these challenges with harm reduction principles to consider patient preferences, decrease length of stay when safe, and support successful completion of antibiotics^1^. We aimed to describe antibiotic course outcomes 4 years after implementation of OPTIONS-DC.

**Methods:**

We conducted a retrospective review of patients who had an OPTIONS-DC from February 2018 to February 2022. Any PWUD diagnosed with a serious infection requiring > 2 weeks of IV antibiotic treatment were eligible for an OPTIONS-DC. Data was collected via a REDCap database. R version 3.3.2 (R Core Team, 2016) was used for statistical analysis.

**Results:**

144 conferences were included, for 129 unique patients. The mean age was 39.4 with 56.3% being male. The most common infections were bacteremia (52.8%) endocarditis (36.1%), and osteomyelitis (47.9%) with a mean recommended treatment duration of 5.62 weeks (Table 1). 87 (60.4%) patients with any substance use initiated medication assisted therapy (MAT) during admission including 74.5% (79/106) of those with opioid use disorder. 112 (77.8%) antibiotic courses were completed with 71 (63.4%) completed outpatient. Of those who initiated or continued MAT during admission, 83 (74.1%) completed antibiotic therapy. Course completion was similar for those discharged home at 84% (21/25) or to a Skilled Nursing Facility (SNF) 81.8% (9/11). All 18 patients who received antibiotics via home infusion vendor and 17 (81%) via infusion center completed their course. 39 (39.8%) patients received long acting IV antibiotics and 23 (23.5%) received oral antibiotics to complete their course. Average out of hospital antibiotic days for those who completed their course were 30.5.

**Table 1.**
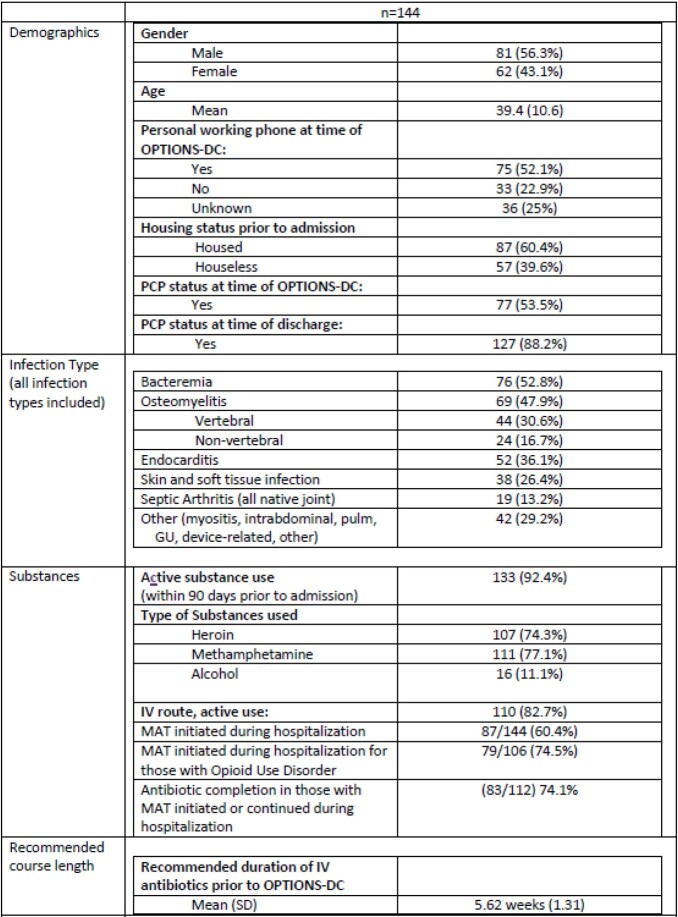
OPTIONS-DC Table 1

**Table 1.**
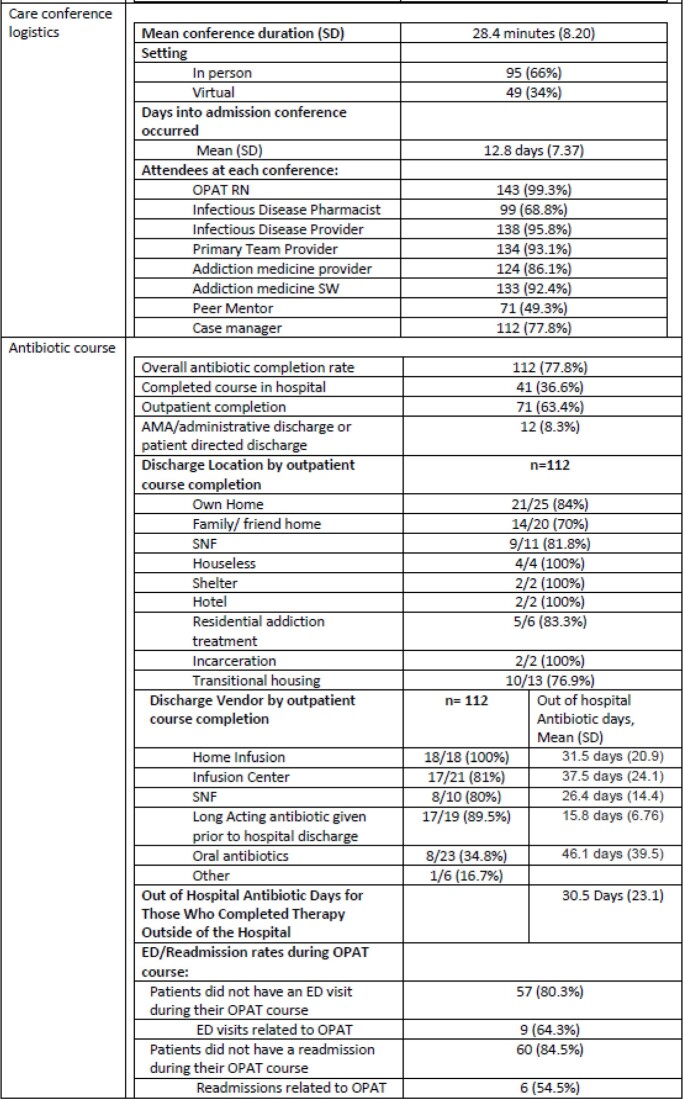
OPTIONS-DC Table 1 continued

**Conclusion:**

The OPTIONS-DC model continues to be a useful tool by incorporating harm reduction principles and patient preferences for PWUD at our hospital. Our completion rate of 77.8% and outpatient completion rate of 63.4% are higher while the patient directed discharge rate of 8.3% is lower than what is generally reported in literature for this population^2-5^.

**Disclosures:**

**Monica K. Sikka, MD**, F2G: Site research investigator.

